# Comments on Paper Published in IJPH as “Factors Associated with Physical Activity among Macedonian Adolescents ...”

**Published:** 2017-02

**Authors:** Erfan AYUBI, Kamyar MANSORI

**Affiliations:** 1. Dept. of Epidemiology, School of Public Health, Shahid Beheshti University of Medical Sciences, Tehran, Iran; 2. Dept. of Epidemiology and Biostatistics, School of Public Health, Tehran University of Medical Sciences, Tehran, Iran; 3. Social Determinants of Health Research Center, Kurdistan University of Medical Sciences, Sanandaj, Iran; 4. Department of Epidemiology, School of Public Health, Iran University of Medical Sciences, Tehran, Iran

## Dear Editor in Chief

We were interested to read the paper by Gontarev S and colleagues that published in the Apr 2016 issue Iran J Public Health (1). The authors aimed to evaluate the effect of demographic, psychological, social and environmental factors with physical activity among Macedonian adolescents from Albanian ethnic community from 11 to 14 yr. the analysis was compared between male and female gender. Their study have demonstrated that mean (SD) of physical activity in male and female were 2.95 (0.67) and 2.75 (0.65), *P-*value<0.001 respectively (1)

Although the statistical method is correct and data are interesting but some methodological and statistical issues should be considered to avoid misinterpretation. Although the mean difference of physical activity between male and female was statistically significant but it is important to emphasize that clinically this mean difference is nothing and clinically negligible. Clinically importance and statistically significant are two differently concepts and as a rule of thumb, clinical importance takes priority over statistical significance. Large ample size, big difference between two means and high variation of the variable in the study population can easily change p-value from non-significant to significant (2).

As the authors point out in their conclusion, Boys unlike girls show significantly higher levels of physical activity, readers should consider the clinical judgments in interpreting of results.
